# Red meat consumption, incident CVD and the influence of dietary quality in the Jackson Heart Study

**DOI:** 10.1017/S1368980022001434

**Published:** 2023-03

**Authors:** Sherman J Bigornia, Sabrina E Noel, Caitlin Porter, Xiyuan Zhang, Sameera A Talegawker, Teresa Carithers, Adolfo Correa, Katherine L Tucker

**Affiliations:** 1 Department of Agriculture, Nutrition, and Food Systems, University of New Hampshire, Durham, NH 03824, USA; 2 Department of Biomedical and Nutritional Sciences, University of Massachusetts Lowell, Lowell, MA, USA; 3 Department of Exercise and Nutrition Sciences, Milken Institute School of Public Health, George Washington University, Washington, DC, USA; 4 Department of Applied Gerontology, School of Applied Science, University of Mississippi, University, MS, USA; 5 Department of Medicine, University of Mississippi Medical Center, Jackson, MS, USA

**Keywords:** Red meat, Processed meat, CVD, Dietary quality, Jackson Heart Study, African Americans

## Abstract

**Objectives:**

We investigated the prospective associations between meat consumption and CVD and whether these relationships differ by dietary quality among African American (AA) adults.

**Design:**

Baseline diet was assessed with a regionally specific FFQ. Unprocessed red meat included beef and pork (120 g/serving); processed meat included sausage, luncheon meats and cured meat products (50 g/serving). Incident total CVD, CHD, stroke and heart failure were assessed annually over 9·8 years of follow-up. We characterised dietary quality using a modified Healthy Eating Index-2010 score (m-HEI), excluding meat contributions.

**Setting:**

Jackson, MS, USA.

**Participants:**

AA adults (*n* 3242, aged 55 y, 66 % female).

**Results:**

Mean total, unprocessed red and processed meat intakes were 5·7 ± 3·5, 2·3 ± 1·8 and 3·3 ± 2·7 servings/week, respectively. Mostly, null associations were observed between meat categories and CVD or subtypes. However, greater intake of unprocessed red meat (three servings/week) was associated with significantly elevated risk of stroke (hazard ratio = 1·43 (CI: 1·07,1·90)). With the exception of a more positive association between unprocessed meat consumption and stroke among individuals in m-HEI Tertile 2, the strength of associations between meat consumption categories and CVD outcomes did not differ by m-HEI tertile. In formal tests, m-HEI did not significantly modify meat–CVD associations.

**Conclusions:**

In this cohort of AA adults, total and processed meat were not associated with CVD outcomes, with the exception that unprocessed red meat was related to greater stroke risk. Dietary quality did not modfiy these associations. Research is needed in similar cohorts with longer follow-up and greater meat consumption to replicate these findings.

The consumption of unprocessed red meat and processed meat is widely considered to adversely affect risk for cardiometabolic diseases. Federal and professional dietary recommendations support moderation of meat intake as part of a healthy diet^(1,2)^. Considering the long latencies of cardiometabolic diseases, prospective observational cohort studies have been a critical tool in identifying the potential impact of diet on long-term health^(3)^. Meta-analyses of prospective cohort studies provide support that greater intake of unprocessed red and processed meat may increase the risk for type 2 diabetes^(4,5)^, as well as for certain cancers^(6)^.

The impacts of unprocessed red and processed meat consumption on heart disease and stroke, the first and fifth leading causes of US mortality^(7)^, are also of great public health interest. Several recent meta-analyses of prospective cohort studies have examined unprocessed or processed meat consumption with incident CHD^(8,9)^, stroke^(8–11)^ and heart failure^(8,12)^. Greater intakes of both unprocessed red and processed meat have been consistently associated with an elevated risk of stroke^(8–11)^. Lower unprocessed red and processed meat intakes were associated with lower CHD risk in one meta-analysis^(9)^, but not in another^(8)^. Heart failure was reported to be associated with processed meat intake in two meta-analyses^(8,12)^, but conflicting results were observed for unprocessed red meat^(12)^. The low availability of large prospective studies on meat intake in relation to incident CHD^(13–15)^ and heart failure^(16–19)^ may contribute to the inconsistent evidence. Furthermore, evidence from randomised controlled trials support that unprocessed red meat consumption does not adversely affect CVD risk factors, specifically blood lipids and blood pressure^(20)^. This may be because red meat is a source of nutrients that are associated with better cardiometabolic risk^(21–24)^ including vitamin B_6_, vitamin B_12_, Zn and Mg^(25)^.

Further, few studies have been conducted among non-European ancestry cohorts and, specifically, African American (AA) adults. This reduces the generalisability of this growing body of research. AA adults experience higher rates of CVD, compared to Hispanic and non-Hispanic White adults; a disparity expected to be sustained for the next 20 years^(26)^. The prevalence of CVD also varies geographically, with the highest CVD mortality observed in Southern states^(27)^. Some data suggest that, compared to non-Hispanic White adults, AA consume more processed meat, less unprocessed red meat^(28,29)^ and less lean beef^(30)^. AA adults living in the South also tend to have higher total red meat intake than AA adults in other geographic regions^(31)^. A Southern dietary pattern has been characterised by relatively high intake of processed meats, organ meats, fried foods and sugar-sweetened beverages, and low intake of fruit, vegetables and fibre^(32)^. In the REGARDS study, greater adherence to a Southern dietary pattern was associated with CHD^(32)^ and stroke risk^(33)^. Despite the interest in the impact of meat consumption on cardiovascular health and the elevated risk of CVD among AA adults, few studies have examined these diet–disease associations. An analysis from the Black Women’s Health Study reported that total, processed and unprocessed red meat consumption was associated with greater CVD mortality ^(31)^. Further research is necessary to confirm these findings.

Another important question is the potential modifying effect of overall dietary quality on unprocessed red and processed meat associations with CVD outcomes. Greater unprocessed red and processed meat intakes have been correlated with lower overall dietary quality^(34,35)^, suggesting that observed adverse associations with cardiovascular health may be partly due to the lower dietary quality of individuals who have greater meat intake^(35,36)^. We know of only one study to examine this, where greater adherence to the Danish Dietary Guidelines did not modify the adverse associations observed between red meat and processed meat consumption and IHD among Danish adults ^(37)^. In that study, stroke and congestive heart failure were not investigated.

To address limitations of the available evidence, we evaluated prospective associations of total meat, unprocessed red meat and processed meat intakes with CVD (stroke, myocardial infarction or congestive heart failure) in a Southern cohort of AA adults residing in the Jackson, Mississippi area, using data from the Jackson Heart Study (JHS) ^(38)^. We also assessed the potential modifying effect of overall dietary quality on these associations. We hypothesised that adverse associations between meat intake categories and incident CVD, CHD, stroke, and heart failure would be observed, and that they would be stronger with lower overall dietary quality.

## Methods

### Study population

Data are from participants of the JHS, a population-based longitudinal cohort of 5306 non-institutionalised AA adults living in the Jackson, Mississippi area, aged ≥ 21 years. Baseline recruitment occurred between late 2000 and early 2004 from the Jackson site of the Atherosclerosis Risk in Communities study^(39)^ and from resident volunteers, randomly contacted individuals, and secondary family members living in the Jackson Mississippi metropolitan area^(38,40,41)^. For the current analysis, we excluded individuals with baseline CVD, and those missing CVD outcome (CHD, stroke and heart failure), food-frequency data, or any data on control variables, as discussed below. In addition, participants with estimated total energy intake of less than 600 kcal/d or greater than 5000 kcal/d were not included. The final analytical sample size was 3242 men and women. All participants provided written informed consent.

### Unprocessed red and processed meat intake ascertainment

Dietary intake data were collected using the Delta NIRI (Nutrition Intervention Research Initiative) JHS FFQ. This FFQ contains 158 items, was administered by JHS clinic staff and has been previously validated against multiple 24-h recalls and biomarkers for use in this population^(42,43)^. Total meat intake was further categorised into unprocessed red meat (beef and pork) and processed meat (online Supplementary Table 1). Nutrition Data Systems for Research (NDSR, Minneapolis, MN) was used to estimate food and nutrient intakes from FFQ responses. This software also allowed for the weight estimation of meat found in mixed component foods/dishes, such as hamburgers. A weighted value was used to separate the contribution of mixed-meat dishes (i.e. pasta and rice dishes and pizza) to unprocessed red meat and processed meat food categories. Due to the format of the FFQ, processed meats could not be separated into beef and pork. Total meat was calculated as the sum of unprocessed red meat and processed meat intakes. A serving of unprocessed red meat was defined as 120 g, and of processed meat, 50 g^(9)^. Meat intakes were adjusted for total energy using the nutrient density approach and expressed per 2000 kcal. The primary exposures were total meat, unprocessed red meat and processed meat. Unprocessed beef and pork were examined as secondary exposures.

### Dietary quality

Overall dietary quality was measured using the Healthy Eating Index (HEI)-2010 score, which was informed by recommendations from the 2010 Dietary Guidelines^(44)^. Higher HEI-2010 score has been related to lower CVD mortality among AA adults living in the South^(45)^. Further, in relation to CVD mortality, the HEI score (hazard ratio (HR) = 0·74 (CI: 0·69, 0·81) and 0·77 (CI: 0·71, 0·84)), for men and women, respectively, top *v*. bottom quintile) performed similarly to the alternative HEI-2010 (HR = 0·79 (CI: 0·73, 0·86) and 0·76 (CI: 0·69, 0·83)), alternative Mediterranean diet (HR = 0·79 (CI: 0·72, 0·86) and 0·81 (CI: 0·74, 0·89)) and DASH dietary indices (HR = 0·83 (0·76, 0·91) and 0·78 (CI: 0·71, 0·85)) in a large multiethnic cohort study^(46)^. HEI components include total fruit, whole fruit, total vegetables, greens and beans, whole grains, dairy, total protein foods, seafood and plant proteins, fatty acids, refined grains, Na, and empty calories. Refined grains, Na and empty calories are moderation components (limit consumption) and were reversed scored with lower intake relating to higher component score. Components were scored on a 0- to 5-point scale or 0- to 10-point scale, where intermediate values received a proportional score. The overall HEI score was derived by summing the component scores. In our study, total meat intake was inversely associated with dietary quality (rho = –25, *P* < 0·0001). Because multiple components of the HEI score are influenced by meat intake, the main explanatory variable in the present study, we created a modified HEI-2010 score (m-HEI) excluding contributions from processed and unprocessed meat.

Unprocessed red meat and processed meat contribute to multiple components of the HEI score, including total protein, unsaturated fatty acid-to-SFA ratio, Na and empty calories. To derive the m-HEI, processed meat and unprocessed red meat were removed from self-reported intakes of foods. The modified estimated weights of consumed foods were then used as inputs into NDSR to calculate nutrient and food group intakes. Some studies have used HEI cut-offs to indicate a good diet (HEI > 80), a diet that needs improvement (HEI 51 to 80), and a poor diet (HEI < 51)^(47)^. In the present study, 81 individuals were categorised as having a good diet, and 533 as a poor diet, whereas the majority were in the needs improvement category (*n* 2628). Due to the small number of individuals in the good diet category, we chose to categorise JHS participants by lower, medium and higher diet quality using m-HEI tertiles.

### CVD ascertainment

Primary outcomes were total CVD, CHD, stroke and congestive heart failure^(48)^. Annual phone calls to living participants or their proxies were conducted to assess CVD event status. Medical records were reviewed to verify diagnoses. For each hospitalisation or death due to CVD, medical records were obtained. Trained clinicians adjudicated CVD events following published guidelines^(49)^. Ascertainment of heart failure outcomes began on 1 January 2005. Events were available through 2010. Censoring occurred at death, loss-to-follow-up or at the end of the follow-up period.

### Assessment of covariates

Data were obtained through in-home interview and clinic examination. Sociodemographics variables, lifestyle behaviours and medical history were captured by interviewer-administered questionnaires during the in-home visit. Anthropometry, blood sampling and medication use were obtained at the clinic examination. The JHS Physical Activity Survey quantified duration, frequency and intensity of physical activity across four domains: active living, work, home life and sports/exercise activities^(50)^. Minutes per week (min/week) of reported moderate or vigorous physical activity were used to categorise participants according to the American Heart Association’s Life’s Simple 7 metric: poor (0 min/week), intermediate (> 0 to < 150 min/week) or ideal (≥ 150 min/week)^(51,52)^. Smoking status was determined by affirmative responses to the questions: ‘Have you smoked more than 4000 cigarettes in your lifetime?’ and ‘Do you smoke cigarettes?’ Waist circumference, in cm, was the average of two measurements obtained at the umbilicus in the standing position^(53)^.

### Statistical analyses

SAS version 9.4 was used for all analyses. Age- and sex-adjusted demographics and dietary intakes were reported by m-HEI tertile using ANCOVA (proc GLM). *P*_for trend_ was determined by treating m-HEI tertile category as a ordinal variable. Cox proportional hazards (proc PHREG) was used to quantify associations between meat exposures and each CVD outcome. The proportional hazards assumption for Cox regression was examined by inspection of Kaplan–Meier curves and Schoenfeld residual plots for categorical and continuous covariates, respectively. Non-proportional hazards were observed for high school education level and diabetes status. Interaction terms with time to event for these covariates were included in all models. Compared to models without covariates, model fit statistics (–2 log-likelihood, Akaike information criterion and Schwarz’s Bayesian criterion) improved with the addition of selected confounders. Standard errors are presented for mean values and 95 % CI for estimated HR.

In primary analyses, we quantified the associations of total meat (sum of unprocessed red and processed meat), unprocessed red meat and processed meat with CVD, CHD, stroke and heart failure. Models were adjusted for age, sex, high school education attainment, medical insurance, waist circumference, physical activity level, current smoking status, diabetes history, total energy intake and overall dietary quality (m-HEI score). Interaction terms for (1) time to event and high school education attainment and (2) time to event and diabetes history were also added to models. Based on our *a priori* hypothesis, we also stratified our analyses by lower, medium and higher dietary quality accordingly, by m-HEI tertile. Formal tests of effect modification were conducted; interaction terms (e.g. unprocessed red meat × m-HEI) were added to models including variables for meat intake (e.g. unprocessed red meat), dietary quality (m-HEI) and other model covariates. Significant evidence of effect modification was considered at *P* < 0·1. Informed by the results of our primary analyses, we secondarily investigated the individual associations of unprocessed beef and pork with incident stroke in both unstratified and m-HEI-stratified analyses.

## Results

### Sample characteristics

Our cohort sample (*n* 3242) was 66·3 % female with mean age 54·6 ± 0·2 years. Participants with greater unprocessed or processed meat intake, tended to be younger, male, and have higher waist circumference, and were less likely to have an ideal physical activity level (Table 1). The HEI score tended to be lower with greater meat consumption, as were the subcategories of total fruit, whole fruit, whole grains, dairy, seafood and plant protein, and unsaturated-to-saturated fat ratio. Further, both unprocessed red and processed meat intakes were associated with greater Na and empty calorie intakes. Mean total meat, unprocessed red meat and processed meat intakes were 5·7 ± 0·06, 2·3 ± 0·03, and 3·3 ± 0·05 servings/week, respectively. Mean unprocessed red meat intakes ranged from 0·8 ± 0·03 to 4·2 ± 0·03 servings/week across consumption tertiles, whereas processed meat intake ranged from 1·2 ± 0·05 to 6·1 ± 0·05 servings/week. (Table 2).

**Table 1:** Baseline sample characteristics by unprocessed red meat and processed meat consumption*

Sample characteristics				Unprocessd red meat (tertiles)										Processed meat (tertiles)						
	1			2			3			*P*_for trend_	1			2			3			*P*_for trend_
	*n* 1080			*n* 1081			*n* 1081				*n* 1080			*n* 1081			*n* 1081			
	%	Mean	se	%	Mean	se	%	Mean	se		%	Mean	se	%	Mean	se	%	Mean	se	
Age, years		57·6	0·36		55·2	0·36		50·9	0·36	<0·0001		56·7	0·37		54·5	0·37		52·5	0·37	<0·0001
Male	26·9			32·1			42·2			<0·0001	30·4			35·1			35·6			0·01
High school education or equivalent	84·2				83·4			82·5		0·29		86·2			82·8			81·1		0·0008
Medical insurance	88				87·1			87·3		0·61		86·4			88·9			87·1		0·62
Waist circumference, cm		98·6	0·49		99·7	0·48		102	0·49	<0·0001		97·9	0·48		101	0·48		102	0·48	<0·0001
Physical activity level†																				
Poor	41·1			47·4			50·9			<0·0001	42·2			47·4			49·9			0·0003
Intermediate	34·6			33			32·5			0·30	34			34			32			0·31
Ideal	24·3			19·6			16·6			<0·0001	23·8			18·6			18·1			0·001
Current smoker	8·84			10·6			12·2			0·01	9·46			10·4			11·7			0·10
Diabetes	15·6			16·6			18·1			0·13	12·2			17·3			20·7			<0·0001
Hypertension	51·3			51·7			54·4			0·14	51			53·4			53			0·32
																				
Dietary variables‡																				
Total energy, kcal		1960	26		2050	26		2210	26	<0·0001		1970	26		2090	26		2170	26	<0·0001
HEI-2010 score		60·9	0·29		58·8	0·29		58	0·29	<0·0001		61·0	0·29		59·1	0·29		57·7	0·29	<0·0001
Total fruit, cup		1·5	0·02		1·3	0·02		1·1	0·02	<0·0001		1·5	0·02		1·2	0·02		1·1	0·02	<0·0001
Whole fruit, cup		0·7	0·02		0·6	0·01		0·6	0·01	<0·0001		0·7	0·01		0·6	0·01		0·6	0·01	<0·0001
Total vegetables, cups		1·1	0·01		1·2	0·01		1·2	0·01	<0·0001		1·2	0·02		1·2	0·01		1·2	0·01	0·53
Green vegetables and beans, cups		0·3	0·01		0·3	0·01		0·4	0·01	0·01		0·3	0·01		0·3	0·01		0·3	0·01	0·81
Whole grains, oz		1·5	0·03		1·0	0·03		0·9	0·03	<0·0001		1·3	0·03		1·1	0·03		1·0	0·03	<0·0001
Dairy, cups		1·1	0·02		1·0	0·02		0·9	0·02	<0·0001		1·1	0·02		1·0	0·02		0·9	0·02	<0·0001
Total protein, oz		5·0	0·06		5·7	0·06		7·0	0·06	<0·0001		5·2	0·06		5·8	0·06		6·7	0·06	<0·0001
Seafood and plant protein, oz		2·2	0·04		2·0	0·04		1·8	0·04	<0·0001		2·3	0·04		2·0	0·04		1·7	0·04	<0·0001
Unsaturated-to-saturated fat ratio		2·1	0·01		2·0	0·01		1·9	0·01	<0·0001		2·1	0·01		2·0	0·01		1·9	0·01	<0·0001
Refined grains, oz		4·5	0·04		4·6	0·04		4·5	0·04	0·26		4·4	0·04		4·6	0·04		4·6	0·04	0·003
Na, grams		3·7	0·03		3·9	0·03		4·1	0·03	<0·0001		3·6	0·03		3·9	0·03		4·3	0·03	<0·0001
Empty calories (solid fats, added sugar, alcohol), % kcal		27·7	0·26		27·7	0·25		26·4	0·20	0·0006		27·9	0·26		27·6	0·25		26·3	0·25	<0·0001

* Means ± se or proportions, adjusted for sex and age (as appropriate) and stratified by unprocessed red and processed meat intake tertiles.

† Physical activity level was defined according to American Heart Association criteria.

‡ Daily nutrient and food intakes are expressed per 2000 kcal unless otherwise noted. Diet data were collected using a validated semi-quantitative FFQ.

**Table 2:** Meat intake by unprocessed red meat and processed meat intake*

Meat, servings/week†	Unprocessed red meat (tertiles)						Processed meat (tertiles)					
	1		2		3		1		2		3	
	*n* 1080		*n* 1081		*n* 1081		*n* 1080		*n* 1081		*n* 1081	
	Mean	se	Mean	se	Mean	se	Mean	se	Mean	se	Mean	se
Total meat	3·6	0·09	5·2	0·09	8·2	0·09	3·0	0·07	5·2	0·07	8·9	0·07
Unprocessed red meat	0·8	0·03	1·9	0·03	4·2	0·03	1·8	0·05	2·4	0·05	2·8	0·05
Beef	0·6	0·03	1·4	0·03	3·14	0·03	1·4	0·04	1·8	0·04	1·9	0·04
Pork	0·2	0·02	0·5	0·02	1·05	0·02	0·4	0·02	0·6	0·02	0·8	0·02
Processed meat	2·7	0·08	3·3	0·08	4·0	0·08	1·2	0·05	2·7	0·05	6·1	0·05

* Mean ± se adjusted for age, sex and total energy intake using ANCOVA.

† A serving was defined as 4·2 oz (120 g) for unprocessed red meat and 1·8 oz (50 g) for processed meat. Diet data were collected using a validated semi-quantitative FFQ.

Participants with greater m-HEI score tended to be older, more physically active, female, to have attained at least a high school education and to have health insurance. They were also more likely to have diabetes or hypertension (online Supplemental Table 2). As expected, m-HEI score was positively associated with HEI adequacy scores for component foods and nutrients (fruit, vegetables, whole grains, dairy, total and seafood and plant protein, unsaturated-to-saturated fat ratio) and with lower intake of moderation components (refined grains, Na and empty calories) (Table 1). Total meat, unprocessed red meat and processed meat consumption were significantly higher among those in the middle m-HEI tertile, relative to the bottom or top tertiles (*P* < 0·05) (online Supplemental Table 3).

### Meat food sources

The top five contributors to total meat consumption were ground beef (15·8 %), luncheon meats (14·3 %); chicken-fried steak (11·2 %), pork main dishes (11·1 %), and hot dogs and sausages (10·5 %) (online Supplementary Table 1). These foods accounted for 62·9 % of total meat intake.

### Total, unprocessed red and processed meat consumption and incident CVD

CVD incidence rates/1000 person-years (P-Y) were 10·2 (CVD), 3·8 (CHD), 2·6 (stroke) and 5·9 (heart failure). Total, unprocessed red and processed meat intakes were not significantly associated with all CVD, CHD or heart failure (Table 3). However, each three serving/week intake of unprocessed red meat consumption was associated with 42 % higher risk of stroke (HR = 1·43, CI: 1·07, 1·90). Conversely, null associations were observed between total and processed meat consumption and stroke. In analyses stratified by m-HEI score tertile, total and processed meat intakes were not significantly associated with CVD or CVD type (CHD, stroke and HF) (*P* > 0·05, for all) (Table 4). Unprocessed red meat was also not associated with most CVD outcomes, with the exception of of stroke among those in the m-HEI Tertile 2 (HR = 2·45, CI: 1·32, 4·55). Formal tests of effect modification did not support that associations of total meat, unprocessed red meat and processed red meat with CVD, CHD, stroke and heart failure were modified by m-HEI score (*P*_for interaction_ ≥ 0·1 for all, data not shown).

**Table 3:** Associations between meat consumption and incidence of CVD†

Meat, three servings/week‡	Full sample	
	*n* 3242	
	HR	95 % CI
All CVD (cases/1000 P-Y)	10·2	
Total meat	1·00	0·91, 1·11
Unprocessed red meat	1·06	0·86, 1·29
Processed meat	0·98	0·86, 1·12
CHD (cases/1000 P-Y)	3·8	
Total meat	1·02	0·87, 1·19
Unprocessed red meat	1·11	0·82, 1·50
Processed meat	0·95	0·77, 1·18
Stroke (cases/1000 P-Y)	2·6	
Total meat	0·94	0·77, 1·16
Unprocessed red meat	1·43	1·07, 1·90*
Processed meat	0·71	0·50, 1·00
Heart failure (cases/1000 P-Y)	5·9	
Total meat	1·04	0·92, 1·17
Unprocessed red meat	0·99	0·76, 1·3
Processed meat	1·05	0·90, 1·21

HR, hazard ratio; P-Y, person-years.

* *P* < 0·05.

† Covariates include baseline sex and baseline values for age, high school attainment, medical insurance, smoker, waist circumference, diabetes status, physical activity level, as well as, total energy and modified HEI-2010 score. Values are HR (95 % CI) and can be interpreted as the increase in risk associated with each three serving/week increase in the meat exposure of interest.

‡ A serving was defined as 4·2 oz (120 g) for unprocessed red meat and 1·8 oz (50 g) for processed meat. Diet data were collected using a validated semi-quantitative FFQ.

**Table 4:** Associations between meat consumption and incidence of CVD stratified by modified HEI score†

Meat, 3 servings/2000 kcal/week‡	Modified HEI-2010 score, tertile					
	1		2		3	
	*n* 1080		*n* 1081		*n* 1081	
	HR	95 % CI	HR	95 % CI	HR	95 % CI
All CVD (cases/1000 P-Y)	10·4		9·3		10·8	
Total meat	0·97	0·81, 1·16	1·04	0·87, 1·24	1·03	0·87, 1·22
Unprocessed red meat	0·99	0·67, 1·47	1·18	0·81, 1·73	1·06	0·77, 1·45
Processed meat	0·94	0·75, 1·19	1·00	0·79, 1·26	1·01	0·82, 1·26
CHD (cases/1000 P-Y)	3·1		4·2		4·2	
Total meat	0·94	0·65, 1·35	0·98	0·75, 1·28	1·11	0·87, 1·41
Unprocessed red meat	0·93	0·45, 1·91	1·15	0·65, 2·04	1·19	0·78, 1·81
Processed meat	0·92	0·55, 1·48	0·91	0·62, 1·31	1·03	0·74, 1·43
Stroke (cases/1000 P-Y)	3·2		1·9		2·8	
Total meat	0·77	0·51, 1·16	1·20	0·84, 1·73	0·94	0·67, 1·32
Unprocessed red meat	1·07	0·53, 2·17	2·45	1·32, 4·55*	1·40	0·94, 2·08
Processed meat	0·64	0·35, 1·19	0·82	0·44, 1·52	0·66	0·37, 1·17
Heart failure (cases/1000 P-Y)	5·60		5·80		6·50	
Total meat	1·04	0·85, 1·28	1·02	0·82, 1·28	1·05	0·85, 1·30
Unprocessed red meat	1·10	0·66, 1·85	0·91	0·55, 1·52	1·00	0·65, 1·56
Processed meat	1·02	0·79, 1·31	1·08	0·82, 1·41	1·06	0·81, 1·38

HR, hazard ratio; P-Y, person-years.

* *P* < 0·05.

† Covariates include baseline sex and baseline values for age, high school attainment, medical insurance, current smoker, waist circumference, physical activity level, diabetes status and total energy. Values are HR (95 % CI) and can be interpreted as the increase in risk associated with each three serving/week increase in the meat exposure of interest.

‡ A serving was defined as 4·2 oz (120 g) for unprocessed red meat and 1·8 oz (50 g) for processed meat. Diet data were collected using a validated semi-quantitative FFQ.

In secondary analyses, we explored whether the association of unprocessed red meat with stroke was driven by beef or pork consumption. We observed that beef (HR = 1·45 (1·07, 1·96)), but not pork (HR = 1·26 (0·44, 3·61)) was significantly associated with stroke overall, and among those in m-HEI Tertile 2 (HR = 3·00 (1·38, 6·52)) (online Supplementary Table 4).

## Discussion

Contrary to our hypothesis, after 9·8 years of follow-up, meat consumption (total meat, unprocessed red meat and processed meat) was not significantly associated with incident CVD or CVD types (CHD, stroke and heart failure), with the exception of unprocessed red meat, particularly beef, on stroke. Further, our results do not support that overall dietary quality, as measured by the m-HEI, differentially impacts associations of these meat categories with incident CVD or examined CVD subtypes.

To our knowledge. only one other study has investigated the prospective associations between red and processed meat consumption and CVD outcomes, specifically among AA adults. Using 22 years of follow-up data from the Black Women’s Health Study, a large cohort of AA women living across the USA, Sheehy et al^(31)^ found that each serving per d increase in unprocessed red and processed red meat intakes was associated with 9 % (HR = 1·09 (CI: 1·00, 1·18)) and 14 % (1·09 (CI: 1·07, 1·21)) greater risk of CVD mortality, respectively. Although CVD mortality was not examined in the present study, we observed that neither CVD (non-fatal and fatal) nor CHD was associated with unprocessed red meat or processed meat intake. For comparisons purposes, our observed HR in servings/d of unprocessed red meat with CVD and CHD were 1·13 (0·71,1·83) and 1·11 (0·82,1·50), respectively. For processed meat, these values were 0·96 (0·71,1·29) and 0·95 (0·77,1·18), respectively. Our study builds upon prior evidence in AA adults^(31)^ by examining individual CVD events, including CHD, stroke and heart failure, as well as total CVD.

Few prospective cohort studies have investigated unprocessed red and processed meat intakes with CHD^(13–15)^ or heart failure^(16–19)^. We observed that neither unprocessed red nor processed meat intakes were related to CHD. This is consistent with evidence from a multiethnic US cohort study (about 25 % Black)^(14)^ and a recent meta-analysis of prospective cohorts^(8)^. In contrast, processed meat was associated with increased risk of CHD among US female nurses^(15)^ and Danish men^(13)^. Our findings that unprocessed red meat was not associated with heart failure is in line with results from two Swedish cohort studies^(17,18)^ and a recent meta-analysis^(12)^. Contradicting our null observations between processed meat and heart failure, these same studies reported an adverse association^(12,17,18)^.

We may have observed null associations in our study, in part, because unprocessed red and processed meat consumption in the JHS did not reach threshold levels required to adversely affect CVD risk. Trends in US meat consumption have been estimated using data from NHANES cycles 1999–2000 to 2015–2016.^(54)^ During this time period, mean intakes for adult groups 20 years and older ranged from 11·7 to 12 oz/week (284 to 340 g/week) for unprocessed red meat and 6·4 to 6·9 oz/week (182 to 196 g/week) for processed meat^(54)^. In the JHS, reported mean intakes per 2000 kcal were lower, at 9·7 oz/week for unprocessed red meat and 6·0 oz/week for processed meat. Low variability in meat intake in JHS may have also contributed to the observed null associations. Another potential reason for null results could have been the relatively short duration of follow-up (9·8 years). In a meta-analysis of prospective cohort studies, greater risk of CVD mortality from unprocessed red meat intake was observed among studies with 15 years or more follow-up, but not in those followed for less than 15 years^(55)^.

The use of a single FFQ collected at baseline prevented us from examining changes in dietary intake during follow-up that may have an impact on CVD risk. This may introduce misclassification errors related to meat intake. The FFQ used in the JHS did not allow for the separation of processed meat by type (red meat *v*. poultry). In another study, red processed meat, specifically, was adversely associated with CHD risk^(13)^. Additionally, the JHS FFQ could not discern between most dishes prepared at home and pre-prepared frozen dishes. The latter may contain additives that may influence CVD risk, including preservatives. As reviewed elsewhere, there is great heterogeneity in how meat is defined across research studies^(56)^. Given the paucity of studies examining unprocessed red and processed meat consumption among AA adults and of studies examining CHD and heart failure, further studies examining CVD subtypes are needed with longer duration of follow-up, repeated measures of dietary intake and conducted in communities with greater variation in meat intake.

A critical barrier to improving dietary recommendations for unprocessed red meat and processed meat consumption for CVD risk reduction is the need for a clearer understanding of the impact of overall dietary quality on these relationships. Although we observed that unprocessed red meat intake was associated with greater risk of stroke among participants categorised as having medium dietary quality (m-HEI Tertile 2), tests of effect modification were not significant. Thus, the present study does not support that overall dietary quality influences the associations of unprocessed red and processed meat intake with CVD, CHD, stroke and heart failure. It is possible that our observation of a lack of effect modification by HEI score was due to a low variability in HEI scores. Our results are, however, in support of those from a recent study using data from the Danish National Survey on Diet and Physical Activity^(37)^. Dietary quality measured using a Danish Dietary Guidelines score did not significantly modify the associations of unprocessed red and processed meat intakes and IHD. In contrast to the present study, they did not exclude contributions from meat intake in their dietary quality score. Other studies have examined the potential modifying effect of fruit and vegetable intake on unprocessed red and processed meat and CVD associations^(36,57)^. In a cohort of Swedish adults, the harmful associations between red meat consumption and CVD mortality were not modified by fruit and vegetable consumption^(36)^.

In the present study, unprocessed meat consumption was associated with a significantly greater risk of stroke. This result, however, should be interpreted with caution as it may be a chance finding due to multiple testing. Our findings are inconsistent with results from randomised controlled trials showing that red meat consumption does not adversely affect blood pressure^(20,58)^, a leading risk factor for stroke^(59)^. Regardless, unprocessed red meat consumption could specifically elevate stroke risk through several biological mechanisms. Some epidemiological evidence supports that greater Fe status^(60)^ and hereditary hemochromatosis^(61)^ are risk factors for stroke. It has been proposed that Fe-catalysed reactions may result in thrombus formation^(62,63)^, which can contribute to ischemic stroke. An estimated 87 % of strokes are ischemic^(64)^. Beef is the third major source of dietary Fe in the USA^(65)^, and our results suggest that unprocessed beef was the main driver of the unprocessed meat–stroke association. In addition, unprocessed meats, particularly those that are cooked through direct heat (e.g. frying), are a major source of dietary advanced glycation end products^(66)^. In the present study, chicken-fried steak and ground beef, the latter typically consumed as hamburgers, were the top two contributors to unprocessed beef consumption. Dietary advanced glycation end products are generated through the Maillard reaction and have proinflammatory properties, including activation of the NF-κβ pathway^(67)^. Advanced glycation end product biomarkers are associated with an elevated risk of stroke^(68)^, as well as CHD^(68)^ and peripheral arterial disease^(69)^. In addition, meals containing fresh red meat may be a source of Na. Na in the form of salt is commonly added to red meat as well as to foods often consumed with red meat, such as French fries. Salt reduction reduces blood pressure^(70)^ and elevated salt consumption increases the risk for stroke^(59)^.

Our study has several strengths. We carefully considered the effect of overall dietary quality on associations between meat consumption and CVD risk. We conducted this study in an AA adult cohort, a group that experiences disproportionate CVD burden. We also considered a number of potential confounders. A limitation of the present study is the relatively low number of observed events, which resulted in wide confidence limits, lowering our ability to detect associations. In the ARIC study, CHD incidence rates/1000 P-Y were reported to be 5·1 and 10·6 among Black women and men, respectively^(71)^, whereas it was 3·8 in the present study. Repeating analyses after a longer duration of follow-up will help to address this issue. Other limitations include the lack of objective biomarkers of meat consumption and the potential for residual confounding. The present study examined data from individuals living in Jackson, Mississippi. The average overall dietary quality (HEI-2010) in the present study (60·3 ± 11 for women and 57·4 ± 10 for men) was comparable to that reported in another study of AA adults living in the South (60·0 ± 12 for women and 55·3 ± 11)^(45)^. However, the limited geographic representation of the present study reduces the generalisability of our findings. Although a semi-quantitative FFQ validated for use among AA adults was used to measure long-term dietary intakes, the self-reported nature of the assessment method increases the chance for non-differential misclassification. This issue may have contributed to the null findings.

Among AA adults followed for 9·8 years, we observed that total meat, unprocessed red meat and processed meat intakes were generally not associated with elevated risk of CVD, CHD, stroke or heart failure. Unprocessed red meat, in particular beef, was associated with increased risk of stroke. There was little evidence to support that consuming a healthier overall diet impacted the strength of these associations. Additional studies in AA adult cohorts of longer duration follow-up and with greater meat intake are needed to replicate these findings.

## Supplementary material

For supplementary material accompanying this paper visit https://doi.org/10.1017/S1368980022001434. click here to view supplementary material
