# Supplementary material for: Red meat consumption, incident CVD and the influence of dietary quality in the Jackson Heart Study
Source: Public Health Nutr. 2022 Jun 23;26(3):643–52. doi: 10.1017/S1368980022001434 (PMC9989707; doi:10.1017/S1368980022001434)
Supplement: Supplementary file 1 [file S1368980022001434sup.zip › S1368980022001434sup002.docx]

| **Supplementary Table 1. Percent contribution of meat food sources to total meat consumption by modified HEI 2010 score** | | | | |
| --- | --- | --- | --- | --- |
| Total meat components | Full sample | Modified HEI 2010 Score, Tertile | | |
|  |  | 1 | 2 | 3 |
| *Unprocessed Beef* |  |  |  |  |
| Chicken-fried steak | 11.2 | 12.3 | 10.9 | 10.4 |
| Ground beef (including from hamburgers) | 15.8 | 17.0 | 16.1 | 14.2 |
| Beef main dishes | 9.9 | 9.0 | 9.6 | 11.5 |
| Beef stews, pies, and soups | 3.4 | 3.1 | 3.4 | 3.9 |
| *Unprocessed Pork* |  |  |  |  |
| Cracklings | 2.1 | 1.9 | 2.0 | 2.6 |
| Pork main dishes (includes ground pork) | 11.1 | 10.5 | 11.8 | 10.9 |
| Neck bones, ham hocks, and pig's feet | 3.4 | 3.5 | 3.1 | 3.5 |
| *Processed meat* |  |  |  |  |
| Cured, lard and salt pork | 2.4 | 2.3 | 2.3 | 2.8 |
| Bacon and breakfast sausage | 5.9 | 5.9 | 5.9 | 5.9 |
| Hot dogs and sausage | 10.5 | 10.3 | 10.7 | 10.5 |
| Lunch meat (includes smoked ham) | 14.3 | 14.2 | 14.1 | 14.7 |
| *Mixed meat dishes* |  |  |  |  |
| Pasta dishes (including spaghetti and lasagna) | 3.3 | 3.3 | 3.4 | 3.3 |
| Pizza | 3.4 | 3.6 | 3.6 | 2.8 |
| Meat and rice dishes | 3.1 | 3.2 | 3.1 | 3.0 |
| Values represent the percent contribution of individuals foods to energy intake from total meat consumption. | | | | |

| **Supplementary Table 2. Baseline sample characteristics by modified HEI 2010 score**^†^ | | | | |
| --- | --- | --- | --- | --- |
|  | Modified HEI 2010 Score, Tertile | | | *P* trend |
|  | 1 | 2 | 3 |  |
|  | n=1080 | n=1081 | n=1081 |  |
| Age, years | 52.6 ± 0.4 | 54.3 ± 0.4 | 56.9 ± 0.4 | <0.0001 |
| Male, % | 37 | 35.8 | 28.3 | <0.0001 |
| High School Education or equivalent, % | 78.1 | 83.8 | 88.2 | <0.0001 |
| Medical insurance, % | 83.6 | 87.9 | 90.9 | <0.0001 |
| Waist circumference, cm | 99.5 ± 0.5 | 101 ± 0.5 | 101 ± 0.5 | 0.12 |
| Physical Activity Level^‡^ |  |  |  |  |
| Poor, % | 52.5 | 48.2 | 38.7 | <0.0001 |
| Intermediate, % | 31.9 | 32.7 | 35.4 | 0.08 |
| Ideal, % | 15.5 | 19.1 | 25.8 | <0.0001 |
| Current smoker, % | 14.5 | 10.3 | 6.77 | <0.0001 |
| Diabetes, % | 13.2 | 15.4 | 21.6 | <0.0001 |
| Hypertension, % | 48 | 53.6 | 55.7 | 0.0001 |
|  |  |  |  |  |
| *Dietary variables*^§^ |  |  |  |  |
| Total Energy, kcal | 2360 ± 25 | 2110 ± 25 | 1760 ± 27 | <0.0001 |
| Modified HEI 2010 score | 50.0 ± 0.14 | 61.9 ± 0.14 | 72.8 ± 0.14 | <0.0001 |
| HEI 2010 score | 48.5 ± 0.15 | 59.3 ± 0.15 | 70.1 ± 0.15 | <0.0001 |
| Total fruit, cups | 0.9 ± 0.02 | 1.23 ± 0.03 | 1.6 ± 0.03 | <0.0001 |
| Whole fruit, cups | 0.5 ± 0.01 | 0.5 ± 0.02 | 0.7 ± 0.02 | <0.0001 |
| Total vegetables, cups | 0.9 ± 0.01 | 1.1 ± 0.01 | 1.3 ± 0.01 | <0.0001 |
| Green vegetables and beans, cups | 0.2 ± 0.01 | 0.31 ± 0.01 | 0.4 ± 0.01 | <0.0001 |
| Whole grains, oz | 0.6 ± 0.03 | 0.9 ± 0.02 | 1.7 ± 0.03 | <0.0001 |
| Dairy, cups | 0.9 ± 0.02 | 1.0 ± 0.02 | 1.1 ± 0.02 | <0.0001 |
| Total Protein, oz | 5.1 ± 0.07 | 6.1 ± 0.06 | 6.4 ± 0.07 | <0.0001 |
| Seafood and plant protein, oz | 1.3 ± 0.04 | 1.9 ± 0.04 | 2.6 ± 0.04 | <0.0001 |
| Unsaturated to saturated fat ratio | 1.8 ± 0.01 | 1.9 ± 0.01 | 2.2 ± 0.01 | <0.0001 |
| Refined grains, oz | 4.9 ± 0.04 | 4.7 ± 0.04 | 3.8 ± 0.05 | <0.0001 |
| Sodium, grams | 4.1 ± 0.03 | 4.0 ± 0.04 | 3.5 ± 0.04 | <0.0001 |
| Empty calories (solid fats, added sugar, alcohol), % kcal | 33.9 ± 0.22 | 26.2 ± 0.21 | 21.7 ± 0.22 | <0.0001 |
| ^†^Means ± SE or proportions, adjusted for sex and age (as appropriate) and stratified by modified Healthy Eating Index 2010 score Tertile. The modified HEI 2010 score was calculated after excluding contributions from red and processed meat including that from mixed dishes such as from pizza and hamburgers.  ^‡^Physical Activity Level was defined according to American Heart Association criteria.  ^§^Daily nutrient and food intakes are expressed per 2000 kcals unless otherwise noted. | | | | |

| **Supplementary Table 3. Dietary consumption by modified HEI 2010 score**^†^ | | | | |
| --- | --- | --- | --- | --- |
| **Meat intake, servings/week** | Modified HEI 2010 Score, Tertile^‡^ | | | *P* trend |
|  | 1 | 2 | 3 |  |
|  | n=1080 | n=1081 | n=1081 |  |
| Total Meat | 5.5 ± 0.10^a^ | 6.1 ± 0.10^b^ | 5.4 ± 0.10^a^ | 0.94 |
| Unprocessed Red Meat | 2.2 ± 0.05^a^ | 2.5 ± 0.05^b^ | 2.2 ± 0.05^a^ | 0.91 |
| Beef | 1.7 ± 0.04^a^ | 1.8 ± 0.04^b^ | 1.7 ± 0.04^a^ | 0.86 |
| Pork | 0.6 ± 0.02^a^ | 0.7 ± 0.02^b^ | 0.6 ± 0.02^a^ | 0.93 |
| Processed Meat | 3.2 ± 0.08^a^ | 3.6 ± 0.08^b^ | 3.2 ± 0.08^a^ | 0.98 |
| ^†^Values are means ± SE, adjusted for sex, age, and total energy.  ^‡^The modified HEI 2010 score was calculated after excluding contributions from unprocessed red and processed meat including that from mixed components foods/dishes such as pizza and hamburgers. ANCOVA was used to estimate meat consumption within m-HEI tertiles. Tukey’s post-hoc analyses were conducted to make paired comparisons. Means with different letter are significantly different from each other (P < 0.05). | | | | |

| **Supplementary Table 4. Associations between unprocessed beef and pork consumption and incidence of stroke stratified by modified HEI score**^†^ | | | | |
| --- | --- | --- | --- | --- |
| Meat, 3 servings/week^‡^ | Modified HEI 2010 Score, Tertile | | | Total Sample |
|  | 1 | 2 | 3 |  |
|  | n=1080 | n=1081 | n=1081 | n=3242 |
|  | HR (95% CI) | HR (95% CI) | HR (95% CI) | HR (95% CI) |
| *Cases/1000 P-Y* | *3.2* | *1.9* | *2.8* | *2.6* |
| Unprocessed beef | 1.15 (0.497, 2.65) | 3.00 (1.38, 6.52)* | 1.40 (0.923, 2.13) | 1.45 (1.07, 1.96)* |
| Unprocessed pork | 0.789 (0.090, 6.88) | 1.75 (0.474, 6.45) | 1.37 (0.205, 9.13) | 1.26 (0.437, 3.61) |
|  |  |  |  |  |
| ^†^Model covariates include baseline sex and baseline values for age, high school attainment, medical insurance, smoker, waist circumference, physical activity level, diabetes status, and total energy. Values are hazard ratios (95% CI) and can be interpreted as the increase in risk associated with a 3 serving/wk increase in the meat exposure of interest. *P<0.05. P-Y, Person-Years.  ^‡^A serving was defined as 4.2 oz (120 g) for unprocessed red meat and 1.8 oz (50 g) for processed meat. | | | | |
